# Mi Vida Saludable: Content Validity and Reliability of The Preferences and Self-Efficacy of Diet and Physical Activity Behaviors Questionnaire for Latina Women (PSEDPALW) for Cancer Survivors

**DOI:** 10.3390/nu15163563

**Published:** 2023-08-12

**Authors:** Pamela A. Koch, Rachel Paul, Isobel R. Contento, Heewon L. Gray, Amanda M. Marín-Chollom, Margarita Santiago-Torres, Hanjie Shen, Salene M. W. Jones, Dawn L. Hershman, Heather Greenlee

**Affiliations:** 1 Department of Health and Behavior Studies, Teachers College, Columbia University, New York, NY 10027, USA; rachelcpaul@gmail.com (R.P.); irc6@tc.columbia.edu (I.R.C.); 2 College of Public Health, University of South Florida, Tampa, FL 33612, USA; hlgray@usf.edu; 3 Department of Psychological Science, Central Connecticut State University, New Britain, CT 06050, USA; amarin-chollom@ccsu.edu; 4 Fred Hutchinson Cancer Center, Seattle, WA 98109, USA; msantiag@fredhutch.org (M.S.-T.); hshen2@fredhutch.org (H.S.); smjones3@fredhutch.org (S.M.W.J.); hgreenlee@fredhutch.org (H.G.); 5 College of Physicians and Surgeons, Mailman School of Public Health, Columbia University, New York, NY 10027, USA; dlh23@cumc.columbia.edu

**Keywords:** theory-based determinants, breast cancer survivors, Latina, instrument validity testing, instrument reliability testing

## Abstract

The purpose of this study is to conduct validity and reliability testing of a new instrument, the Preferences and Self-Efficacy of Diet and Physical Activity Behaviors Questionnaire for Latina Women (PSEDPALW)*,* which is for women who identify as Latina and are breast cancer survivors. PSEDPALW measures preferences and self-efficacy for four behaviors: physical activity (PA), fruit and vegetable (FV) intake, dietary fat (DF) intake, and added sugar (AS) intake (eight scales in total). Validity testing was conducted through an expert panel review and a cognitive interviewing focus group (*n* = 4). Reliability was tested via internal consistency reliability (*n* = 118) and test–retest reliability (*n* = 30). Validity testing was used to refine PSEDPALW. Reliability testing was conducted on three versions with 104, 47, and 41 items. PA scales had acceptable Cronbach’s α (>0.70) but low ICC (NS). FV and DF scales had acceptable Cronbach’s α (>0.70), with preferences for the shorter (47- and 41-item) versions (Cronbach’s α < 0.70), and all scales had moderate ICC (*p* < 0.05, except the FV scale on the 104-item version (*p* = 0.07)). The AS preferences scale had Cronbach’s α < 0.70, with self-efficacy > 0.70 for all versions and ICC moderate for all versions (*p* ≤ 0.01). PSEDPALW may be useful to assess diet and physical activity preferences and self-efficacy in theory-based diet and physical activity interventions in women who identify as Latina and are breast cancer survivors.

## 1. Introduction

Breast cancer incidence rates are high; in 2023, nearly 298,000 women are expected to be diagnosed with breast cancer, and about 43,700 women will die of breast cancer in the U.S. [1]. More than 3.8 million women have a history of breast cancer and are either in treatment or posttreatment. Following a healthy diet and engaging in regular physical activity improves morbidity and mortality in breast cancer and other chronic diseases [2,3,4,5]. Recommendations on post-diagnosis diet and physical activity by the World Cancer Institute/American Institute for Cancer Research (AICR) and the American Cancer Society (ACS) include being physically active and consuming a diet high in fruits and vegetables and low in energy-dense foods, i.e., foods high in fat and sugar [6,7].

Yet, most breast cancer survivors do not meet these recommendations; most women fall short of the recommended daily five or more servings of fruits and vegetables and consume more calories from solid fat, alcohol, and added sugar than recommended [8,9]. Only 40% of cancer survivors have a high-quality diet [10]. Further, fewer than half of breast cancer survivors meet the physical activity recommendations of 150 min or more of moderate to vigorous activity per week [11,12].

Women who identify as Latina when compared to women who identify as Caucasian and not Latina have disparities in access to health care, higher rates of diabetes and obesity, lower rates of physical activity, and worse morbidity and mortality [13,14,15,16,17,18]. While women who identify as Latina have lower incidence of breast cancer than woman who identify as Caucasian and not Latina, they are more likely to be diagnosed with invasive breast cancer at a younger age and at an advanced stage, and they are more likely to die younger than women who identify as Caucasian [19,20].

Current practices to educate cancer patients on diet and physical activity recommendations typically include a brief clinician consultation and/or providing print information (e.g., brochures or a summary care plan) [21,22]. However, behaviorally focused, theory-based, and culturally tailored education interventions consistently show higher effectiveness in changing behavior compared to most current techniques [22,23,24,25,26]. A challenge to conducting research to determine the effectiveness of educational interventions is a lack of instruments to measure psychosocial determinants that motivate and facilitate behavior change. These psychosocial theory-based determinants are often mediators of behavior change; they include self-efficacy (confidence in changing behavior), perceived benefits (of changing behavior), and preferences (liking the behavior, such as liking specific vegetables). There is a particular lack of measurement instruments in cancer survivor and Latino populations. Yet, without such measurements, it is not possible to know if changes in behavioral outcomes are due to changes in psychosocial determinants. Further, these psychosocial measures are needed to identify which determinants are the most important to target in future interventions. 

A recent systematic review of psychosocial theories used in the development and evaluation of dietary interventions for people who have survived cancer found that the use of theories was common in the development of the interventions. However, in instruments used to evaluate outcomes, measuring psychosocial theory-based determinants was rare. More specifically, the 19 studies identified in this review used one or more psychosocial theories to guide the intervention, with social cognitive theory (SCT) [27], transtheoretical theory [28], and the theory of planned behavior [29] being the most common. Yet, measuring theory-based determinants as outcomes was only done in 4 of 19 studies (21%) [30]. Of these four studies, three measured self-efficacy and three measured readiness to change [30]. Thus, there is a need for valid and reliable instruments to measure key theory-based determinants in studies that evaluate the outcomes of theory-based interventions.

To date, the majority of diet and physical activity intervention studies with female breast cancer survivors have been based on SCT, which posits that behavioral, personal, and environmental factors synergistically determine behavior [27]. Interventions among cancer survivors based on SCT have significantly improved weekly physical activity and fruit and vegetable intake [25,31,32,33,34,35,36]. Among SCT’s personal factors (that is, people’s beliefs, values, and interactions with other people), the determinants of preferences and self-efficacy were found to effectively mediate behavior change in breast cancer survivors participating in our prior randomized, controlled trial, ¡Cocinar Para Su Salud! (Cook For Your Health!), which tested an experiential group-based intervention focused on increasing fruit and vegetable intake and decreasing fat intake [25,37] in Latina breast cancer survivors (R21CA152903, PI: H Greenlee). Our subsequent trial, Mi Vida Saludable (My Healthy Life) tested in-person and digital interventions based on ¡Cocinar Para Su Salud! but expanded to address four behavior change goals in Latina breast cancer survivors: (1) increase physical activity, (2) increase fruit and vegetable intake, (3) decrease dietary fat intake, and (4) decrease added sugar intake (R01CA186080, PI: H Greenlee). Mi Vida Saludable interventions were delivered in Spanish to breast cancer survivors as part of a randomized, controlled trial to determine the separate and synergistic effects of theory-based classroom and electronic nutrition and physical activity education [38].

We developed the **P**references and **S**elf-**E**fficacy of **D**iet and **P**hysical **A**ctivity Behaviors Questionnaire for **L**atina **W**omen (PSEDPALW) to measure the determinants of preferences and self-efficacy for these four behaviors (a total of eight scales) for use in the Mi Vida Saludable trial. The questionnaire was developed in English and translated into Spanish. We tested validity and reliability on the Spanish version. PSEDPALW focuses on four behavior change goals (increasing physical activity, increasing fruit and vegetable intake, decreasing dietary fat intake, and decreasing added sugar intake) and two theory-based determinants (preferences and self-efficacy).

To measure preferences and self-efficacy, as with any theory-based determinant, we need scales that are valid and reliable through having acceptable internal consistency and test–retest scores, as well as scales that are parsimonious in order to reduce participant burden. We based our measures of preferences and self-efficacy on measures that have previously been found reliable for assessing preferences and self-efficacy for eating fruits and vegetables [39]. We used the style of these measures to create similar items for our other three behaviors. To assess physical activity self-efficacy, we used an instrument previously tested for reliability [40]. For each of the four behaviors, our goal was to develop scales that have acceptable internal consistency and test–retest correlations.

The purpose of this study was to describe the development, content validity testing, and reliability testing of PSEDPALW.

## 2. Materials and Methods

### 2.1. Instrument Development

We developed PSEDPALW by modifying and adapting existing validated questionnaires to the specific population of Latina women and behaviors and that were targeted in the Mi Vida Saludable trial [25]. We considered the demographics of our audience as we developed the first version of PSEDPALW. While the women who participated in the Mi Vida Saludable trial were from diverse national ethnic backgrounds, we knew that the majority of the women would be from the Dominican Republic, based on the demographics of the patient population and recruitment patterns from our previous research. As part of the eligibility criteria, all participants self-identified as Latina upon enrollment. In addition, participants were asked to self-identify their race/ethnicity. We also knew that the majority of the women would be over 50 years old, given the age distribution of women diagnosed with breast cancer. We considered these demographics in both the development of the PSEDPALW questionnaire as well as the Mi Vida Saludable intervention. Similar interventions developed for cancer survivors and/or Latina women that address physical activity as the behavior change goal have addressed the determinants of perceived benefits, perceived barriers, and self-efficacy [40]. Research suggests that the determinants of preferences and self-efficacy are key in changing fruit and vegetable intake, dietary fat intake, and added sugar intake [25,41]. Thus, for the four behavior change goals, we decided to address the two determinants of self-efficacy and preferences. The original PSEDPALW had 8 scales, with a total 104 questions in those 8 scales.

The questionnaire was first developed in English and then was translated into Spanish by a bilingual staff member. The questionnaire was then reviewed by three bilingual team members, with the goal of making the questionnaire appropriate for the intended audience, and finally reviewed for accuracy by a certified bilingual translator. The staff discussed discrepancies in order to reach consensus [42,43].

The Columbia University Medical Center Institutional Review Board approved this study (AAAP0461). The study underwent expedited review. All participants were adults who provided written informed consent. Below, we describe the eight scales included in the 104-item questionnaire.

#### 2.1.1. Physical Activity—Preferences

This 16-item scale was adapted from the National Cancer Institute’s (NCI) Food Attitudes and Behaviors (FAB) Survey. The FAB measures preferences for fruits and vegetables and was shown to be reliable in non-Hispanic adults (*n* = 3397) from the United States [39]. We used the same style of questions to ask about preferences for different physical activities. The physical activities were based on physical activities common to the target population and were discussed and recommended in the Mi Vida Saludable intervention, (e.g., walking at a brisk pace for leisure).

#### 2.1.2. Physical Activity—Self-Efficacy

This 9-item scale was directly adapted from the Exercise and Nutrition Routine Improving Cancer Health (ENRICH) questionnaire [40]. This questionnaire was previously validated with a Canadian adult population (*n* = 703), and the Cronbach’s alphas were α = 0.88 (baseline), α = 0.89 (6 months), and α = 0.90 (12 months) [44].

#### 2.1.3. Fruits and Vegetables—Preferences

This 28-item scale asked about how much participants like or dislike specific fruits and vegetables. This scale was adapted from the NCI FAB [39]. The adaptations that we made were to change the specific vegetables to be those that were cooked, discussed, and encouraged to be eaten in the Mi Vida Saludable intervention. The intervention focused on vegetables, with some that would be familiar to the participants (e.g., calabaza squash, sweet potatoes, and bell peppers) and some that may not be as familiar (e.g., cauliflower, brussels sprouts, and leeks). Since the intervention focused more on vegetables than fruit, our scale did as well. The FAB included 17 fruits, whereas the PSEDPALW included only one fruit (apples), which was included in the FAB. The FAB included 20 vegetables, and the PSEDPALW included 27 vegetables, of which 10 were included in the FAB [39]. 

#### 2.1.4. Fruits and Vegetables—Self-Efficacy

This 7-item scale was based directly on the FAB [39]. The FAB presented different conditions that may make it hard to eat fruits and vegetables (i.e., when hungry; when tired; when junk foods are in the house; eating cookies, candy, ice cream, or other sweet desserts instead of fruits; when friends and family are eating junk foods; eating at work; snacking on junk foods instead of fruits or vegetables while watching television). This scale of 7 items had a Cronbach’s alpha of 0.92 [39].

#### 2.1.5. Dietary Fat Intake—Preferences

This 14-item scale used the same strategy as the fruit and vegetable preferences scale, asking about how much participants liked or disliked the foods low in dietary fat that were common in the target population and would be discussed and cooked during the Mi Vida Saludable intervention.

#### 2.1.6. Dietary Fat Intake—Self-Efficacy

This 14-item scale uses the exact same foods that were asked about in the preferences scale. The style of the questions used the style for self-efficacy questions from the FAB [39], i.e., “assuming you want to, how confident are you that you could choose (e.g., chicken without the skin instead of chicken with skin) starting this week and continuing for at least 1 month”. There were questions about low-fat meat (e.g., poultry instead of red meat, removing visible fat), low-fat dairy (e.g., choosing low-fat dairy and small portions of full-fat dairy), preparation of starchy foods (e.g., baked instead of fried), and low-fat cooking methods (e.g., roasted, baked, broiled, or boiled instead of fried; using cooking oil instead of butter or lard; adding less fat when cooking).

#### 2.1.7. Added Sugar Intake—Preferences

This 8-item scale used the same format as the dietary fat intake preferences scale. We included in the questionnaire the foods recommended for a low added sugar consumption during the Mi Vida Saludable intervention (e.g., water, sparkling water, or water mixed with sliced fruit or vegetables).

#### 2.1.8. Added Sugar Intake—Self-Efficacy

This 8-item scale was paired with the food asked about in the preference questions and used the self-efficacy format adapted from the FAB [39] (i.e., “Assuming you want to, how confident are you that you could choose…”.). There were questions about beverages (e.g., coffee or tea without added sugar), cereal (choosing varieties without added sugar), dessert (e.g., choosing fresh or frozen fruit instead of sweet treat desserts such as cookies, candy, cakes, or pastries), dairy (e.g., choosing yogurt and milk without added sugar), and others (e.g., choosing salad dressings and sauces without added sugar or making your own without added sugar).

### 2.2. Validity Testing: Content Validity and Face Validity

#### 2.2.1. Content Validity: Expert Panel Review

Content validity assesses whether an instrument includes important aspects of the constructs of interest (in this case, important aspects of our determinants) and can be determined by relevant experts reviewing the questions (items) for representativeness of the determinants, readability, clarity, and comprehensiveness [45]. Then, experts determine which items to include in the final questionnaire [45,46]. An expert panel of senior researchers, community leaders, and the study coordinator [A.M.M.C.] reviewed PSEDPALW for accuracy, relevance, and understandability for the intended population. 

#### 2.2.2. Face Validity with Cognitive Interviewing

Face validity means a questionnaire has “face value”; that is, that the item content appears, on the surface, to measure the construct that it is intended to measure, especially to the study participants [45]. Face validity is often paired with content validity [45] and assessed by cognitive interviewing to detect problems respondents may have in understanding survey instructions and items as well as in formulating answers. To assess face validity, we recruited participants for cognitive interviewing from the Columbia University Medical Center. Inclusion criteria were 21 years of age or older, self-identified as Latina, at least 90 days post-treatment for breast cancer, and able to read and write Spanish. The participants received a paper copy of the questionnaire and completed it, which took from 15 to 30 min. Then, participants took part in the cognitive interview, which was conducted as a group and used a verbal probing technique to ask about the understandability and interpretation of each question and whether it seemed relevant to the purpose of the questionnaire. This 45 min focus group was audio-recorded. The focus group was facilitated by a bilingual researcher. The second author, along with an experienced bilingual note-taker, took notes throughout the focus group. Participants received public transit travel reimbursement and a $20 gift card as compensation for their participation.

### 2.3. Reliability Testing: Internal Consistency and Test–Retest

#### 2.3.1. Internal Consistency with Cronbach’s Alpha and Item-to-Total Correlation

Internal consistency reliability tests the homogeneity of the items within a scale that is intended to measure a single phenomenon, in this case, scales for preferences and self-efficacy for each of the 4 behavior change goals (8 total scales) [47]. Internal consistency was assessed with the questionnaires completed by the participants in the Mi Vida Saludable trial, which is described in the Recruitment section below.

We performed statistical analysis using IBM SPSS Statistics, version 20 (SPSS Inc., Chicago, IL, USA, 2012). Internal consistency of each scale was evaluated by calculating Cronbach’s alpha and corrected item-to-total correlations. A Cronbach’s alpha value > 0.70 indicated sufficient internal consistency [48]. For individual items, an item-to-total Pearson correlation of more than 0.40 indicated good reliability to the scale [49]. To create shorter versions to reduce participant burden and increase internal consistency, questions with low item-to-total correlations were eliminated. Cronbach’s alpha and item-to-total correlations were recalculated for the shorter questionnaires. The initial questionnaire is denoted “PSEDPALW-104”. The second iteration is “PSEDPALW-47”, with questions eliminated from all 8 scales. The third iteration is denoted as “PSEDPALW-41”, as another 6 questions were eliminated from the “reducing added sugar” and “reducing fat” subscales.

#### 2.3.2. Test–Retest with Interclass Correlation Coefficients (ICCs)

Test–retest reliability measures the stability of an instrument at 2 time points [47]. Test–retest reliability of the questionnaire was assessed by calculating correlations between T1 and T2 using the intraclass correlation coefficients (ICCs) and was used as the main outcome to determine reliability. The level of agreement of the ICC was categorized as follows: <0.5 = poor; 0.5–<0.75 = moderate; 0.75–<0.90 = good; and >0.90 = excellent [50].

### 2.4. Recruitment

We recruited study participants from the Columbia University Medical Center and breast cancer patient database. Recruitment notices were sent via mailed letters. We also promoted this study at other New York City metropolitan area cancer centers and on the Avon Army of Women community notice website. Finally, we attended and distributed flyers at cancer awareness events throughout New York City.

Potential participants were eligible if they were female, ≥21 years of age, self-identified as Latina or Hispanic, and were able to speak English or Spanish. In addition, they had to self-identify as having a medical history of histologically confirmed stage 0–III breast cancer, with no evidence of metastatic disease, and be ≥90 days post–final treatment, including chemotherapy, biologic therapy, i.e., trastuzumab, radiation therapy, or breast surgery (current use of hormonal therapy was permitted). Participants had to be willing and able to receive emails and text messages and/or attend in-person educational classes and to travel to Columbia University Medical Center for data collection. Participants were ineligible if they smoked. There were also behavioral inclusion criteria. Participants had to consume <5 serving of fruits and vegetables per day, measured by the NIH Fruit and Vegetables Screener [51], and/or engage in <150 weekly minutes of moderate to vigorous physical activity, measured by two questions from the International Physical Activity Questionnaire (IPAQ) short form [52]. Both questionnaires had previously been validated among Hispanic/Latino populations.

In total, we recruited five cohorts of women. The women in the first two cohorts were given the option to complete the questionnaire for test–retest reliability purposes and received an additional $25 gift card as compensation for their participation. The participants who were part of the test–retest completed the questionnaire a second time, one to three weeks after the first administration, with the second administration occurring before the Mi Vida Saludable intervention began. We estimated that, for the test–retest based on having 8 scales to compare, a sample size of 30 was adequate [53]; this was in addition to the general recommendation that a sample size of 30 is adequate for reliability testing [50].

## 3. Results

Table 1 presents the demographic and socioeconomic data for participants who were part of the internal consistency testing, all five cohorts (*n* = 118), and those from the first two cohorts (*n* = 64) who agreed to take part in the test–retest reliability (*n* = 30). The majority of the participants were from the Dominican Republic (73%). Of those that completed the internal consistency testing, almost 80% had a household income ≤ $30,000, whereas this income level comprised 60% of those that did the test–retest reliability; about 57% of both groups were currently on a food assistance program. The percentage of women who had overweight was 43.9% and who had obesity was 40.2% (84.1% total for overweight and obesity). This is similar to the population of Latina women in the United States, of which 31.4% have overweight and 48.9% have obesity (80.3% total for overweight and obesity) [54].

### 3.1. Content Validity—Expert Panel Review

The expert panel review of content resulted in revisions to the questions for clarity, such as additional examples of foods listed in the dietary fat and sugar intake questions. Additionally, changes were made to the layout, such as the addition of pictures of fruits and vegetables, which would help participants identify unfamiliar items, e.g., winter squash. The panel was asked if there were any additional questions that should be included, and the panel agreed there were no questions to add. The panel also agreed that all questions were relevant, and no questions were eliminated. Thus, the overall number of questions stayed the same.

### 3.2. Face Validity—Cognitive Interviewing

While the expert panel included members of the community who reviewed the questions for readability, clarity, and cultural appropriateness, we conducted a focus group cognitive interview (*n* = 4) in Spanish to further assess the participants’ understanding of the questions and their intent [55]. The participants had a mean age of 58 and had a mean of 6.6 years since breast cancer diagnosis. Three out of the four were from the Dominican Republic, and all four participated in the Supplement Nutrition Assistance Program (SNAP) and/or the Women, Infants, and Children (WIC) program, which is a proxy indicator of a low-income population. The review of the transcript from this focus group revealed that the participants had a good understanding of the PSEDPALW questionnaire. Participants understood the questions, layout, and type of answers desired for the PSEDPALW. Participants felt the physical activity questions were clear. Participants accurately explained the questions in their own words, providing evidence that they understood the questions and their intent. They reported the layout and answer options were clear, and they did not have any suggestions for changes.

There was no specific feedback, or suggested edits, on questions related to fruits and vegetables. For questions related to dietary fat, the women wanted to include examples of alternative dairy sources, particularly almond milk, coconut milk, soymilk, and rice milk, which they drank more often than cow’s milk. The question was amended to say “Choose low-fat 1% or non-fat skim milks (cow, almond, soy, rice) instead of cream or 2% or whole milk?” Women liked the combinations of animal proteins that helped them understand the types of food groupings we were aiming for (e.g., “chicken and turkey” and “salami or sausage”). Women also requested fish to be included as a low-fat animal protein example. In addition, women requested “boiling” to be added as a low-fat cooking method. When discussing potatoes, women requested that “red potatoes” be specifically included as a baked option as they frequently consumed these. For questions related to added sugar, some women discussed adding brown sugar instead of white sugar to their beverages, e.g., coffee and tea. The question was broadened to encompass “any kind of added sugar”. They also reported that they felt there were too many questions and that their attention waned towards the end. Since the participants of this focus group had a good understanding of the questionnaire, we did not conduct further cognitive testing. All the suggestions made were incorporated into the final instrument.

The findings from the expert review and cognitive interviewing thus resulted in PSEDPALW-104, with 104 questions across the eight scales (preferences and self-efficacy scales for each of the four categories: physical activity, fruits and vegetables, dietary fat intake, added sugar intake).

### 3.3. Internal Consistency Reliability

To reduce respondent burden, the number of items from each of the scales of the PSEDPALW-104 was reduced to produce additional shorter instruments: a refined scale of 47 items (PSEDPALW-47), and the most parsimonious scale of 41 items (PSEDPALW-41). Table 2 provides internal consistency data from the 118 participants for eight scales for PSEDPALW-104, PSEDPALW-47, and PSEDPALW-41. The physical activity and fruits and vegetables scales were reduced once (PSEDPALW-47), and dietary fat and added sugar twice (PSEDPALW-47 and PSEDPALW-41). PSEDPALW-104 for all eight scales show Cronbach’s alphas ranging from 0.77 to 0.92, which are above the >0.70 acceptable level, with several in the good to excellent range. PSEDPALW-47 and PSEDPALW-41 for physical activity preferences and self-efficacy scales and for fruit and vegetables, dietary fat intake, and sugar intake self-efficacy scales were all above the acceptable level. The scales that showed lower internal consistency with fewer items and that were below the 0.70 Cronbach’s alpha were the preference scales for PSEDPALW-47 and PSEDPALW-41 for fruit and vegetables, dietary fat intake, and added sugar intake. Thus, the scales showed acceptable to excellent internal consistencies, except the preferences scales for the behaviors of fruits and vegetables, dietary fat, and dietary sugar for the shorter versions of the instrument, which did not meet the threshold of Cronbach’s α ≥ 0.70 for acceptable internal consistencies.

### 3.4. Test–Retest Reliability

Summary outcomes of test–retest reliability are presented in Table 3. The participants for this analysis are the 30 women who completed the measures twice, before the intervention was initiated. For the physical activity scales, the ICC agreement indicated poor reliability (0.28–0.33), with the exception of the PSEDPALW-104 self-efficacy scale, which was moderate (0.56). For the fruit and vegetable intake, dietary fat intake, and added sugar intake scales, the ICC agreement was moderate for all scales (0.55–0.72), with the exception of the PSEDPALW-104 self-efficacy scale for added sugar intake, which had good agreement (0.77). Thus, the test–retest reliabilities for the dietary scales were moderate to excellent, while those for physical activity were mostly poor.

## 4. Discussion

The current study contributes to the literature by reporting on the psychometrics of an instrument that addresses two key psychosocial determinants (preferences and self-efficacy) for four important cancer-risk-reducing behavior change goals (increasing physical activity, increasing fruit and vegetable intake, decreasing dietary fat intake, and decreasing added sugar intake). Several studies conducted with people who have survived cancer used only one item to measure a determinant for each behavior, not a scale with several questions as we have done in PSEDPALW, and hence could not report psychometrics with which we could compare [56,57,58,59]. The initial version of PSEDPALW had 104 questions for the eight scales, which we were able to reduce to two shorter versions with either 47 or 41 items, to reduce respondent burden. For a very thorough assessment of the psychosocial determinants, researchers would want to choose the full version with 104 questions. Most researchers will want to choose one of the shorter versions with 47 or 41 questions. For the version with 41 questions, there are fewer questions on dietary fat and dietary sugar. Researchers with a particular focus on those behaviors may want to choose the version with 47 questions. Content validity with expert review and face validity with cognitive interviewing successfully improved the questionnaire before quantitative analysis of reliability.

The internal consistency reliabilities of the eight scales of PSEDPALW are good to moderate and similar to those of existing validated instruments for adults in general [60,61,62,63]. While many studies report psychometric information on self-efficacy scales, reports on preferences in terms of diet in adults are rare, with some reporting measures of enjoyment for physical activity [64]. The internal consistency reliabilities of the physical activity preferences and self-efficacy scales are good and similar to those of the instruments from which they derived, the FAB [39] and ENRICH [44], as well as to several others [60,62,63]. While the full scales had better reliability than the reduced scales, the differences are not substantial. For the fruit and vegetable preferences scale, our full version with 28 items and FAB with 36 items had similar and good Cronbach’s alphas; these are similar to an instrument that measured preferences for fruits and vegetables for 11 and 12 year olds [65] and one for enjoyment of healthy eating in adults [63]. When the number of items was reduced from 28 to 6 in the reduced scales, the Cronbach’s alpha decreased from 0.87 to 0.53, suggesting that more items may be needed to measure preferences. For the fruit and vegetable self-efficacy scales, the full and reduced scales had similar good internal consistencies, which were similar to those reported for several instruments for adults [60,63]. Likewise, for the dietary fat intake preferences scale, the reduced scales did not have acceptable Cronbach’s alphas, whereas all three versions of the self-efficacy scale did. Here the Cronbach’s alphas were similar to a psychosocial measurement tool for adults [63]. For the added sugar intake preferences scale, Cronbach’s alphas were below the acceptable level for all three versions of PSEDPALW, while they were all acceptable for the self-efficacy scale. It appears that in terms of liking or preferences for different foods, scales with more items had higher Cronbach’s alphas, suggesting that measures of preferences need to have an adequate number of items.

The test–retest, intraclass correlations for each of these scales for each of the behaviors were moderate or better for all the scales in all three versions, except for the physical activity preferences scale, which was relatively poor. These results are similar to those in several other studies [60,61,62].

Thus, PSEDPALW is an instrument that is novel, with generally good psychometrics for measuring two key psychosocial determinants for four behaviors important to health: physical activity, fruit and vegetable intake, fat intake, and intake of added sugar. This instrument can be used for studies that aim to improve diet and physical activity for people who have survived cancer, and specifically for women who identify as Latina. While the shorter versions have a somewhat lower reliability for some scales, they have considerably less respondent burden.

Strengths of the current analysis include the development being based on previously validated survey instruments. The physical activity and fruit and vegetable scales were based on previously validated surveys with cancer survivors [40,44] and with an adult population mirroring the US population [39], respectively. The PSEDPALW-47 is brief, takes 10 to 15 min to complete, and is easy to administer.

Consequently, this questionnaire can be used for measuring preferences and self-efficacy among women who identify as Latina breast cancer survivors to examine the impact of interventions on these mediators for physical activity and diet behaviors and to conduct mediational analyses. Further work refining and testing this questionnaire is warranted in similar populations, including other Latina populations and among other cancer survivors.

Our sample was limited to Latina breast cancer survivors residing in an urban, low-income environment and is not representative of all Americans. Our results may not apply to all women who identify as Latina or to all cancer survivors. Preferences and self-efficacy, our key behavior change goals, may differ based on income and socioeconomic status. The study sample size of *n* = 30 is the minimum recommended for test–retest reliability; future studies could replicate our analyses with additional subjects [66]. Our cognitive interviewing involved a small focus group, which may have missed additional information on the instrument. Finally, self-reported data suffer inherent limitations.

Reliability and validity are closely related and are fundamental prerequisites for each other [67]. The findings of this study, through qualitative and quantitative testing, provide evidence for the initial content validity and reliability of PSEDPALW. A reliable questionnaire can help increase the probability of finding significant correlations and differences in a research project [68].

## 5. Conclusions

The Preferences and Self-Efficacy of Diet and Physical Activity Behaviors Questionnaire for Latina Women is a novel tool to measure two key theory-based psychosocial determinants for four recommended behaviors for people who are survivors of cancer. This instrument was developed for women who identify as Latina, which is a group on whom there has been little research in the past. This paper reports on three versions of PSEDPALW, the full version with 104 items, a reduced version with 47 items, and the most parsimonious version with 41 items. Overall, while the full version, across all scales, was the most reliable, the reduced versions performed well for most scales, and their lower respondent burden make them more useful for most settings. Thus, researchers working with people who are survivors of cancer, and specifically women who identify as Latina who are survivors of breast cancer, may choose to use PSEDPALW for their research. Further research on the development of measures for theory-based psychosocial determinants for this and other populations can build on what is reported in this study. The three versions of the PSEDPALW are available. See Supplementary Materials.

## Figures and Tables

**Table 1:** Demographic and socioeconomic characteristics of participants in the Mi Vida Saludable trial who completed measures to assess reliability of the PSEDPALW.

	Participants Providing Data on Internal Consistency Reliability Measures (*n* = 118)	Participants Providing Data on Test–Retest Reliability Measures (*n* = 30)	*p*-Value
**Demographic characteristics**			
Age, years, Mean (SD)	56.4 (10.5)	53.3 (8.8)	0.06
Race, *n* (%)			0.41
Black or African American	28 (23.7%)	10 (33.3%)	
White	32 (27.1%)	9 (30.0%)	
American Indian or Alaska Native	6 (5.1%)	2 (6.7%)	
More than one race	44 (37.3%)	7 (23.3%)	
Missing	8 (6.8%)	2 (6.7%)	
National ethnic background, *n* (%)			0.77
Argentinian	1 (0.9%)	0 (0.0%)	
Colombian	4 (3.4%)	1 (3.3%)	
Cuban	1 (0.9%)	0 (0.0%)	
Dominican	87 (73.7%)	22 (73.3%)	
Ecuadorian	9 (7.6%)	2 (6.7%)	
Salvadorian	1 (0.9%)	0 (0.0%)	
Honduran	2 (1.7%)	0 (0.0%)	
Mexican	8 (6.8%)	0 (0.0%)	
Nicaraguan	5 (4.2%)	0 (0.0%)	
Peruvian	1 (0.9%)	0 (0.0%)	
Puerto Rican	4 (3.4%)	2 (6.7%)	
Other	1 (0.9%)	3 (10.0%)	
Education, *n* (%)			0.29
Less than high school	35 (29.7%)	5 (16.7%)	
High school graduate or GED	31 (26.3%)	8 (26.7%)	
Some college	27 (22.9%)	9 (30.0%)	
College degree or higher	25 (21.2%)	8 (26.7%)	
Employment, *n* (%)			0.98
Full-time	34 (28.8%)	8 (26.7%)	
Part-time	13 (11.0%)	3 (10.0%)	
Retired	26 (22.0%)	6 (20.0%)	
Homemaker	10 (8.5%)	3 (10.0%)	
Unemployed	15 (12.7%)	5 (16.7%)	
Disabled	19 (16.1%)	5 (16.7%)	
Missing	1 (0.9%)	0 (0.0%)	
Annual household income, *n* (%)			<0.01
$0–$15,000	73 (61.9%)	14 (46.7%)	
$15,001–$30,000	20 (17.0%)	4 (13.3%)	
$30,001–$60,000	12 (10.2%)	7 (23.3%)	
$60,001–$100,000	8 (6.8%)	5 (16.7%)	
Missing	5 (4.2%)	0 (0.0%)	
Currently on food assistance program, *n* (%)			0.83
EBT/SNAP/WIC	68 (57.6%)	17 (56.7%)	
**Acculturation characteristics**			
Born in the US, *n* (%)			0.09
Yes	9 (7.6%)	5 (16.7%)	
No	89 (75.4%)	20 (66.7%)	
Missing	20 (17.0%)	5 (16.7%)	
Number of years living in the US, Mean (SD)	25.8 (12.3)	28.56 (11.6)	0.30
Preferred language, *n* (%)			0.18
Spanish	84 (71.0%)	30 (100.0%)	
English	0 (0%)	0 (0.0%)	
Missing	34 (29.0%)	0 (0.0%)	
**Clinical characteristics**			
BMI categories, *n* (%)			0.54
Normal weight, BMI >18.5 to <25	13 (15.9%)	3 (10.0%)	
Overweight, BMI 25 to <30	36 (43.9%)	14 (46.7%)	
Obese, BMI ≥ 30	33 (40.2%)	13 (43.3%)	
Endocrine therapy use at time of enrollment, *n* (%)			0.92
Yes	40 (47.6%)	15 (50.0%)	
No	44 (52.4%)	15 (50.0%)	
Treatment received			
Breast surgery, *n* (%)			NA
Yes	84 (71%)	30 (100.0%)	
No	0 (0%)	0 (0.0%)	
Missing	34 (29.0%)	0 (0.0%)	
Chemotherapy therapy received, *n* (%)			0.95
Yes	60 (50.9%)	15 (50.0%)	
No	55 (46.6%)	14 (46.7%)	
Missing	3 (2.5%)	1 (3.3%)	
Radiation therapy received, *n* (%)			0.70
Yes	51 (43.2%)	17 (56.7%)	
No	65 (55.1%)	13 (43.3%)	
Missing	2 (1.7%)	0 (0.0%)	
Endocrine therapy received, *n* (%)			0.79
Yes	63 (53.4%)	13 (43.3%)	
No	54 (45.8%)	17 (56.7%)	
Missing	1 (0.9%)	0 (0.0%)	
Medical conditions, *n* (%)			
Hypertension	46 (39.0%)	11 (36.7%)	0.93
Diabetes	24 (20.3%)	5 (16.7%)	0.75
Cholesterol problems	38 (32.2%)	7 (23.3%)	0.33
None	31 (26.3%)	8 (26.7%)	1.00
Comorbidity index score (0–20), Mean (SD)	2.01 (2.1)	2.2 (2.3)	0.55

Abbreviations: SNAP, Supplemental Nutrition Assistance Program; WIC, Special Supplemental Program for Women, Infants, and Children.

**Table 2:** Internal consistency reliability measures of the Preferences and Self-Efficacy of Diet and Physical Activity Behaviors Questionnaire for Latina Women (PSEDPALW), *n* = 118.

	Full Scale PSEDPALW-104			Refined Scale PSEDPALW-47			Most Parsimonious Scale PSEDPALW-41		
**Behavior and Determinant**	No. of items	Range of Item–Total Correlation Coefficients	Cron-bach’s α ^3^	No. of items	Range of Item–Total Correlation Coefficients	Cron-bach’s α ^3^	No. of items	Range of Item–Total Correlation Coefficients	Cron-bach’s α ^3^
**Physical Activity**									
**Preferences ^1^** Please indicate which types of physical activity you like to do. e.g., *Walking at a brisk pace for leisure*	16	0.55–0.82	0.92	8	0.56–0.79	0.85	8	0.56–0.79	0.85
**Self-efficacy ^2^** How confident are you that you could participate in regular moderate to vigorous physical activity over the next month? e.g., *When you are a little tired*	9	0.63–0.74	0.86	5	0.70–0.78	0.80	5	0.70–0.78	0.80
**Fruit and Vegetable Intake**									
**Preferences ^1^** For each of the fruits and vegetables listed below, how much do you like or dislike each type of fruit or vegetable? e.g., *Leeks*	28	0.30–0.69	0.87	6	0.25–0.72	0.53	6	0.25–0.72	0.53
**Self-efficacy ^2^** Assuming that you want to, how confident are you that you could do each of the following starting this week and continuing for at least 1 month? e.g., *Eat a healthy snack, like a fruit or a vegetable, when you’re really hungry?*	7	0.55–0.73	0.78	4	0.73–0.79	0.76	4	0.73–0.79	0.76
**Dietary Fat Intake**									
**Preferences ^1^** How much do you like or dislike each type of food or drink listed below? e.g., *Chicken or turkey slices*	14	0.34–0.69	0.77	6	0.54–0.71	0.64	5	0.53–0.79	0.69
**Self-efficacy ^2^** Assuming that you want to, how confident are you that you could do each of the following starting this week and continuing for at least 1 month? e.g., *Choose chicken or turkey slices instead of salami, sausage, or ham?*	14	0.37–0.71	0.87	6	0.58–0.71	0.71	5	0.55–0.82	0.74
**Added Sugar Intake**									
**Preferences ^1^** How much do you like or dislike each type of food or drink listed below? e.g., *Coffee or tea without added sugar*	8	0.43–0.59	0.61	6	0.43–0.61	0.54	4	0.57–0.70	0.54
**Self-efficacy ^2^** Assuming that you want to, how confident are you that you could do each of the following starting this week and continuing for at least 1 month? e.g., *Choose coffee or tea without added sugar instead of coffee or tea with added sugar?*	8	0.60–0.71	0.80	6	0.59–0.76	0.77	4	0.72–0.76	0.72

^1^ Response options: 0 = Never tried it; 1 = Strongly dislike; 2 = Dislike; 3 = Neutral; 4 = Like; 5 = Strongly like. ^2^ Response options: 1 = Not at all confident; 2 = Not very confident; 3 = Neutral, 4 = Very confident, 5 = Extremely confident. ^3^ Cronbach’s α ≥ 0.70 was considered acceptable internal consistency; ≥0.80 to 0.90 = good; and ≥0.90 = excellent.

**Table 3:** Repeated measures (test–retest) reliability of the Preferences and Self-Efficacy of Diet and Physical Activity Behaviors Questionnaire for Latina Women (PSEDPALW), *n* = 30.

	Full Scale PSEDPALW-104				Refined Scale PSEDPAWL-47				Most Parsimonious Scale PSEDPALW-41			
**Behavior and** **Determinant**	No. of items	Correlation (between T1 and T2) Coefficient	Intraclass Correlation Coefficient ^3^ (95% CI)	*p*-value ^4^	No. of items	Correlation (between T1 and T2) Coefficient	Intraclass Correlation Coefficient (95% CI)	*p*-value ^4^	No. of items	Correlation (between T1 and T2) Coefficient	Intraclass Correlation Coefficient (95% CI)	*p*-value ^4^
**Physical Activity**												
Preferences ^1^	16	0.19	0.33 (−0.42–0.68)	0.30	8	0.16	0.28 (−0.51–0.66)	0.39	8	0.16	0.28 (−0.51–0.66)	0.39
Self-efficacy ^2^	9	0.39	0.56 (0.07–0.79)	0.04	5	0.31	0.47 (−0.12–0.75)	0.10	5	0.31	0.47 (−0.12–0.75)	0.10
**Fruit and Vegetable Intake**												
Preferences ^1^	28	0.56	0.72 (0.40–0.86)	<0.01	6	0.38	0.55 (0.05–0.79)	0.04	6	0.38	0.55 (0.05–0.79)	0.04
Self-efficacy ^2^	7	0.34	0.51 (−0.04–0.77)	0.07	4	0.47	0.64 (0.23–0.83)	0.01	4	0.47	0.64 (0.23–0.83)	0.01
**Dietary Fat Intake**												
Preferences ^1^	14	0.54	0.70 (0.37–0.86)	<0.01	6	0.56	0.72 (0.41–0.87)	<0.01	5	0.56	0.72 (0.40–0.86)	<0.01
Self-efficacy ^2^	14	0.52	0.68 (0.33–0.85)	<0.01	6	0.59	0.74 (0.46–0.88)	<0.01	5	0.51	0.67 (0.32–0.85)	<0.01
**Added Sugar Intake**												
Preferences ^1^	8	0.52	0.68 (0.33–0.85)	<0.01	6	0.54	0.70 (0.37–0.86)	<0.01	4	0.52	0.68 (0.33–0.85)	<0.01
Self-efficacy ^2^	8	0.63	0.77 (0.52–0.89)	<0.01	6	0.53	0.69 (0.35–0.85)	<0.01	4	0.46	0.63 (0.22–0.82)	0.01

^1^ Response options: 0 = Never tried it; 1 = Strongly dislike; 2 = Dislike; 3 = Neutral; 4 = Like; 5 = Strongly like. ^2^ Response options: 1 = Not at all confident; 2 = Not very confident; 3 = Neutral, 4 = Very confident, 5 = Extremely confident. ^3^ ICC levels of agreement: <0.50 = poor; 0.50 ≤ 0.75 = moderate; 0.75 ≤ 0.90 = good; and >0.90 = excellent. ^4^ *p*-value for significance of correlation coefficient between T1 and T2.

## Data Availability

The data presented in this study are available on request from the corresponding author (P.A.K.) and principal investigator (H.G.). The data are not publicly available due to privacy issues.

## Supplementary Materials

The following supporting information can be downloaded at: https://www.mdpi.com/article/10.3390/nu15163563/s1, Questionnaire S1: PSEDPALW-104; Questionnaire S2: PSEDPALW-47; Questionnaire S3: PSEDPALW-41.
