# Supplementary material for: Mi Vida Saludable: Content Validity and Reliability of The Preferences and Self-Efficacy of Diet and Physical Activity Behaviors Questionnaire for Latina Women (PSEDPALW) for Cancer Survivors
Source: Nutrients. 2023 Aug 12;15(16):3563. doi: 10.3390/nu15163563 (PMC10457843; doi:10.3390/nu15163563)
Supplement: Supplementary file 1 [file nutrients-15-03563-s001.zip › nutrients-2531389-supplementary.pdf]

Preferences and Self-Efficacy of Diet and Physical Activity Behaviors Questionnaire for Latina Women – Full Version 104 questions

Section 1. Physical Activity (25 questions)

We will ask you about your physical activity. Please respond to each item by marking ONE box per row with an X.

A. Please indicate which types of physical activity you like to do.

	Strongly dislike	Dislike	Neutral	Like	Strongly like	Never tried it
1. Walking at a brisk pace for leisure	1☐	2☐	3☐	4☐	5☐	6☐
2. Doing housework with vigorous effort	1☐	2☐	3☐	4☐	5☐	6☐
3. Biking using light to moderate effort for leisure	1☐	2☐	3☐	4☐	5☐	6☐
4. Dancing in a group setting	1☐	2☐	3☐	4☐	5☐	6☐
5. Doing moderate activities at the gym	1☐	2☐	3☐	4☐	5☐	6☐
6. Playing with children with vigorous effort	1☐	2☐	3☐	4☐	5☐	6☐
7. Doing vigorous activities at the gym	1☐	2☐	3☐	4☐	5☐	6☐
8. Walking very quickly and/or uphill for leisure	1☐	2☐	3☐	4☐	5☐	6☐
9. Walking at a brisk pace to get from place to place	1☐	2☐	3☐	4☐	5☐	6☐
10. Bicycling using vigorous effort for leisure	1☐	2☐	3☐	4☐	5☐	6☐
11. Biking using light to moderate effort to get from place to place	1☐	2☐	3☐	4☐	5☐	6☐
12. Dancing at home	1☐	2☐	3☐	4☐	5☐	6☐
13. Biking using vigorous effort to get from place to place	1☐	2☐	3☐	4☐	5☐	6☐
14. Doing housework with moderate effort	1☐	2☐	3☐	4☐	5☐	6☐
15. Walking very quickly and/or uphill to get from place to place	1☐	2☐	3☐	4☐	5☐	6☐

16. Playing with children with moderate effort	1☐	2☐	3☐	4☐	5☐	6☐

Definitions. For the following questions,

- **Moderate to vigorous** physical activities refer to activities that take moderate to vigorous physical effort and make you breathe somewhat harder or much harder than normal. You may have a difficult time having conversations. *Examples include* walking briskly, running, carrying children upstairs or for distances, and doing housework like carrying grocery bags.
- **"Regular"** physical activity means 2 ½ hours (150 minutes) per week. If we think about it on a daily basis, it would be 20 minutes every day or 30 minutes 5 times per week.

Please respond to each item by marking ONE box per row with an X.

B. Please rate your confidence in participating in regular moderate to vigorous physical activity over the next month.

**How confident are you that you could participate in regular moderate to vigorous physical activity over the next month?**	**Not at all confident**	**Not very confident**	**Neutral**	**Very confident**	**Extremely confident**
17. When you are a little tired	1☐	2☐	3☐	4☐	5☐
18. When you are in a bad mood or feeling depressed	1☐	2☐	3☐	4☐	5☐
19. When you have to do it by yourself	1☐	2☐	3☐	4☐	5☐
20. When it becomes boring	1☐	2☐	3☐	4☐	5☐
21. When you can't notice any improvements in your fitness	1☐	2☐	3☐	4☐	5☐
22. When you have many other demands on your time	1☐	2☐	3☐	4☐	5☐
23. When you feel a little stiff or sore	1☐	2☐	3☐	4☐	5☐
24. When the weather is bad	1☐	2☐	3☐	4☐	5☐
25. When you have to get up early to do physical activity, even on weekends	1☐	2☐	3☐	4☐	5☐

Section 2. Fruits and Vegetable Intake (35 questions)

Please respond to each item by marking ONE box per row with an X.

A. For each of the fruits and vegetables listed below, how much you like or dislike each type of fruit or vegetable?

	Strongly dislike	Dislike	Neutral	Like	Strongly like	Never tried it
1. Leeks	1☐	2☐	3☐	4☐	5☐	6☐
2. Avocado	1☐	2☐	3☐	4☐	5☐	6☐
3. Apples	1☐	2☐	3☐	4☐	5☐	6☐
4. Scallions/ green onions	1☐	2☐	3☐	4☐	5☐	6☐
5. Broccoli	1☐	2☐	3☐	4☐	5☐	6☐
6. Green cabbage	1☐	2☐	3☐	4☐	5☐	6☐
7. Carrots	1☐	2☐	3☐	4☐	5☐	6☐
8. Cauliflower	1☐	2☐	3☐	4☐	5☐	6☐
9. Arugula	1☐	2☐	3☐	4☐	5☐	6☐
10. Spaghetti squash	1☐	2☐	3☐	4☐	5☐	6☐
11. Beets	1☐	2☐	3☐	4☐	5☐	6☐
12. Mustard greens	1☐	2☐	3☐	4☐	5☐	6☐
13. Calabaza squash	1☐	2☐	3☐	4☐	5☐	6☐
14. Garbanzo beans (chickpeas)	1☐	2☐	3☐	4☐	5☐	6☐
15. Collard greens	1☐	2☐	3☐	4☐	5☐	6☐
16. Kale	1☐	2☐	3☐	4☐	5☐	6☐
17. Shallots	1☐	2☐	3☐	4☐	5☐	6☐
18. Spinach, baby spinach	1☐	2☐	3☐	4☐	5☐	6☐
19. Tomatoes, tomato paste, canned tomatoes	1☐	2☐	3☐	4☐	5☐	6☐
20. Parsnips	1☐	2☐	3☐	4☐	5☐	6☐
21. Peas	1☐	2☐	3☐	4☐	5☐	6☐
22. Sweet potatoes	1☐	2☐	3☐	4☐	5☐	6☐
23. Turnips	1☐	2☐	3☐	4☐	5☐	6☐
24. Purple cabbage	1☐	2☐	3☐	4☐	5☐	6☐
25. Brussels sprouts	1☐	2☐	3☐	4☐	5☐	6☐
26. Bell pepper	1☐	2☐	3☐	4☐	5☐	6☐
27. Butternut squash	1☐	2☐	3☐	4☐	5☐	6☐
28. Chard	1☐	2☐	3☐	4☐	5☐	6☐

Please respond to each item by marking ONE box per row with an X.

B. Assuming that you want to, how confident are you that you could do each of the following starting this week and continuing for at least 1 month?

	Not at all confident	Not very confident	Neutral	Very confident	Extremely confident	Does not apply
29. Eat a healthy snack, like a fruit or a vegetable, when you're really hungry?	1☐	2☐	3☐	4☐	5☐	6☐
30. Eat healthy foods, like fruits or vegetables, when you are tired?	1☐	2☐	3☐	4☐	5☐	6☐
31. Eat healthy foods, like fruits or vegetables, when there are junk foods in your house like chips, cookies, or candy?	1☐	2☐	3☐	4☐	5☐	6☐
32. Eat fruit instead of cake, cookies, candy, ice cream, or other sweets for dessert?	1☐	2☐	3☐	4☐	5☐	6☐
33. Eat fruits and vegetables when your family and friends are eating junk foods like chips, cookies, or candy?	1☐	2☐	3☐	4☐	5☐	6☐
34. Buy or bring fruits and vegetables to eat at work?	1☐	2☐	3☐	4☐	5☐	6☐
35. Snack on fruits and vegetables rather than on junk foods while watching TV?	1☐	2☐	3☐	4☐	5☐	6☐

Section 3. Dietary Fat Intake (28 questions)

Please respond to each item by marking ONE box per row with an X.

A. Next, how much you like or dislike each type of food or drink listed below?

	Strongly dislike	Dislike	Neutral	Like	Strongly like	Never tried it
1. Chicken or turkey slices	1☐	2☐	3☐	4☐	5☐	6☐
2. Low-fat 1% or non-fat skim milks (cow, almond, soy, rice)	1☐	2☐	3☐	4☐	5☐	6☐
3. Baked (not regular, fried) plantain or potato chips	1☐	2☐	3☐	4☐	5☐	6☐
4. Chicken that is roasted, baked, broiled, boiled, or grilled	1☐	2☐	3☐	4☐	5☐	6☐
5. 90% or 95% lean ground meat or turkey	1☐	2☐	3☐	4☐	5☐	6☐
6. Chicken without the skin	1☐	2☐	3☐	4☐	5☐	6☐
7. Turkey burgers or veggie burgers	1☐	2☐	3☐	4☐	5☐	6☐
8. Low-fat or non-fat yogurt	1☐	2☐	3☐	4☐	5☐	6☐
9. Baked (not fried) plantains or potatoes (white, red, sweet)	1☐	2☐	3☐	4☐	5☐	6☐
10. Pork and/ or beef without visible (white) fat	1☐	2☐	3☐	4☐	5☐	6☐
11. Fish that is baked, broiled, or grilled	1☐	2☐	3☐	4☐	5☐	6☐
12. Foods made with oil instead of butter or lard	1☐	2☐	3☐	4☐	5☐	6☐
13. Foods made with small amounts of fat	1☐	2☐	3☐	4☐	5☐	6☐
14. Foods made with a small amount of cheese	1☐	2☐	3☐	4☐	5☐	6☐

Please respond to each item by marking ONE box per row with an X.

B. Assuming you want to, how confident are you that you could do each of the following starting this week and continuing for at least 1 month?

How confident are you that you could…	Not at all confident	Not very confident	Neutral	Very confident	Extremely confident
15. **Choose chicken or turkey slices** instead of salami, sausage, or ham?	1☐	2☐	3☐	4☐	5☐
16. **Choose low-fat 1% or non-fat skim milks (cow, almond, soy, rice)** instead of cream or 2% or whole milks?	1☐	2☐	3☐	4☐	5☐
17. **Choose baked (not regular, fried) plantain or potato chips** instead of regular plantain or potato chips	1☐	2☐	3☐	4☐	5☐
18. **Choose chicken that is roasted, baked, broiled, boiled, or grilled** instead of fried chicken?	1☐	2☐	3☐	4☐	5☐
19. **Choose 90% or 95% lean ground meat or turkey** instead of less lean, 75% to 85% lean, ground meat or turkey?	1☐	2☐	3☐	4☐	5☐
20. **Choose to eat chicken without the skin** instead of chicken with its skin?	1☐	2☐	3☐	4☐	5☐
21. **Choose turkey burgers or veggie burgers** instead of hamburgers or cheeseburgers?	1☐	2☐	3☐	4☐	5☐
22. **Choose low-fat or non-fat yogurt** instead of regular fat yogurt, sour cream, or whipped cream?	1☐	2☐	3☐	4☐	5☐
23. **Baked (not fried) plantains or potatoes (white, red, sweet)** instead of fried plantains, hash browns, or French fries?	1☐	2☐	3☐	4☐	5☐
24. **Choose pork and/or beef without visible (white) fat** instead of pork and/or beef with visible fat?	1☐	2☐	3☐	4☐	5☐
25. **Choose fish that is baked, broiled, or grilled** instead of fried fish?	1☐	2☐	3☐	4☐	5☐

26. **Choose foods cooked with oil** instead of butter or lard?	1☐	2☐	3☐	4☐	5☐
27. **Choose foods made with small amounts of fat** instead of foods made with a lot of fat?	1☐	2☐	3☐	4☐	5☐
28. **Choose foods made with a small amount of cheese** instead of foods with a lot of cheese?	1☐	2☐	3☐	4☐	5☐

Section 4. Dietary Sugar Intake (16 questions)

Please respond to each item by marking ONE box per row with an X.

A. Next, how much you like or dislike each type of food or drink listed below?

	Strongly dislike	Dislike	Neutral	Like	Strongly like	Never tried it
1. Coffee or tea without added sugar	1☐	2☐	3☐	4☐	5☐	6☐
2. Hot cereal without any type of added sugar like plain oatmeal or plain cream of wheat	1☐	2☐	3☐	4☐	5☐	6☐
3. Cold cereal without any type of added sugar like regular Cheerios	1☐	2☐	3☐	4☐	5☐	6☐
4. Fresh or frozen fruit as a sweet, treat, or dessert	1☐	2☐	3☐	4☐	5☐	6☐
5. Plain yogurt (with or without adding fruit yourself) or "light" yogurt	1☐	2☐	3☐	4☐	5☐	6☐
6. Water, sparkling water, or water mixed with slices of fruits or vegetables	1☐	2☐	3☐	4☐	5☐	6☐
7. Plain, unsweetened, milks (cow, almond, soy, rice)	1☐	2☐	3☐	4☐	5☐	6☐
8. Salad dressings and sauces (such as tomato sauce) with low amounts of added sugar or that you make yourself	1☐	2☐	3☐	4☐	5☐	6☐

Please respond to each item by marking ONE box per row with an X.

B. Assuming you want to, how confident are you that you could do each of the following starting this week and continuing for at least 1 month?

How confident are you that you could…	Not at all confident	Not very confident	Neutral	Very confident	Extremely confident	Does not apply
9. **Choose coffee or tea without added sugar** instead of coffee or tea with added sugar?	1☐	2☐	3☐	4☐	5☐	6☐
10. **Choose hot cereal without any type of added sugar like plain oatmeal or plain cream of wheat** instead of hot cereal with added sugar like Maple and Brown Sugar Oatmeal, Cream of Wheat with added sugar?	1☐	2☐	3☐	4☐	5☐	6☐
11. **Choose cold cereal without any type of added sugar like regular Cheerios** instead of cold cereal with added sugar like Honey Nut Cheerios, Special K with Berries, Granola?	1☐	2☐	3☐	4☐	5☐	6☐
12. **Choose fresh or frozen fruit as a sweet, treat, or dessert** instead of cookies, candy, cakes, or pastries?	1☐	2☐	3☐	4☐	5☐	6☐
13. **Choose plain yogurt (with or without adding fruit) or "light" yogurt** instead of ice cream, yogurt with more added sugar, milkshakes?	1☐	2☐	3☐	4☐	5☐	6☐
14. **Choose water, sparkling water, or water mixed with slices of fruits or vegetables** instead of drinks with added sugar such as flavored waters, sodas, juices, or energy drinks?	1☐	2☐	3☐	4☐	5☐	6☐
15. **Plain, unsweetened, milks (cow, almond, soy, rice)** instead of drinks with added sugar such as chocolate or vanilla milks, flavored waters, sodas, juices, or energy drinks?	1☐	2☐	3☐	4☐	5☐	6☐

16. **Choose salad dressings and sauces (such as tomato sauce) with low amounts of added sugar or that you make yourself** instead of salad dressings and sauces with higher amounts of added sugar?	1☐	2☐	3☐	4☐	5☐	6☐

Preferences and Self-Efficacy of Diet and Physical Activity Behaviors Questionnaire for Latina Women – Full Version 104 questions

Behavior	Questions
Physical Activity	25
Fruit and Vegetables	35
Dietary Fat	28
Dietary Sugar	16
Total	104

Preferences and Self-Efficacy of Diet and Physical Activity Behaviors Questionnaire for Latina Women – Shortened version 1, 47 questions

Section 1. Physical Activity (13 questions)

We will ask you about your physical activity. Please respond to each item by marking ONE box per row with an X.

A. Please indicate which types of physical activity you like to do.

	Strongly dislike	Dislike	Neutral	Like	Strongly like	Never tried it
1. Dancing in a group setting	1☐	2☐	3☐	4☐	5☐	6☐
2. Playing with children with vigorous effort	1☐	2☐	3☐	4☐	5☐	6☐
3. Doing vigorous activities at the gym	1☐	2☐	3☐	4☐	5☐	6☐
4. Walking very quickly and/or uphill for leisure	1☐	2☐	3☐	4☐	5☐	6☐
5. Walking at a brisk pace to get from place to place	1☐	2☐	3☐	4☐	5☐	6☐
6. Bicycling using vigorous effort for leisure	1☐	2☐	3☐	4☐	5☐	6☐
7. Dancing at home	1☐	2☐	3☐	4☐	5☐	6☐
8. Doing housework with moderate effort	1☐	2☐	3☐	4☐	5☐	6☐

Definitions. For the following questions,

- **Moderate to vigorous** physical activities refer to activities that take moderate to vigorous physical effort and make you breathe somewhat harder or much harder than normal. You may have a difficult time having conversations. *Examples include* walking briskly, running, carrying children upstairs or for distances, and doing housework like carrying grocery bags.
- **"Regular"** physical activity means 2 ½ hours (150 minutes) per week. If we think about it on a daily basis, it would be 20 minutes every day or 30 minutes 5 times per week.

Please respond to each item by marking ONE box per row with an X.

B. Please rate your confidence in participating in regular moderate to vigorous physical activity over the next month.

**How confident are you that you could participate in regular moderate to vigorous physical activity over the next month?**	**Not at all confident**	**Not very confident**	**Neutral**	**Very confident**	**Extremely confident**
9. When you can't notice any improvements in your fitness	1☐	2☐	3☐	4☐	5☐
10. When you have many other demands on your time	1☐	2☐	3☐	4☐	5☐
11. When you feel a little stiff or sore	1☐	2☐	3☐	4☐	5☐
12. When the weather is bad	1☐	2☐	3☐	4☐	5☐
13. When you have to get up early to do physical activity, even on weekends	1☐	2☐	3☐	4☐	5☐

Section 2. Fruits and Vegetable Intake (10 questions)

Please respond to each item by marking ONE box per row with an X.

A. For each of the fruits and vegetables listed below, how much you like or dislike each type of fruit or vegetable?

	Strongly dislike	Dislike	Neutral	Like	Strongly like	Never tried it
1. Green cabbage	1☐	2☐	3☐	4☐	5☐	6☐
2. Spaghetti squash	1☐	2☐	3☐	4☐	5☐	6☐
3. Kale	1☐	2☐	3☐	4☐	5☐	6☐
4. Tomatoes, tomato paste, canned tomatoes	1☐	2☐	3☐	4☐	5☐	6☐
5. Butternut squash	1☐	2☐	3☐	4☐	5☐	6☐
6. Chard	1☐	2☐	3☐	4☐	5☐	6☐

Please respond to each item by marking ONE box per row with an X.

B. Assuming that you want to, how confident are you that you could do each of the following starting this week and continuing for at least 1 month?

	Not at all confident	Not very confident	Neutral	Very confident	Extremely confident	Does not apply
7. Eat a healthy snack, like a fruit or a vegetable, when you're really hungry?	1☐	2☐	3☐	4☐	5☐	6☐
8. Eat healthy foods, like fruits or vegetables, when you are tired?	1☐	2☐	3☐	4☐	5☐	6☐
9. Eat fruits and vegetables when your family and friends are eating junk foods like chips, cookies, or candy?	1☐	2☐	3☐	4☐	5☐	6☐
10. Snack on fruits and vegetables rather than on junk foods while watching TV?	1☐	2☐	3☐	4☐	5☐	6☐

Section 3. Dietary Fat Intake (12 questions)

Please respond to each item by marking ONE box per row with an X.

A. Next, how much you like or dislike each type of food or drink listed below?

	Strongly dislike	Dislike	Neutral	Like	Strongly like	Never tried it
1. Baked (not regular, fried) plantain or potato chips	1☐	2☐	3☐	4☐	5☐	6☐
2. Chicken that is roasted, baked, broiled, boiled, or grilled	1☐	2☐	3☐	4☐	5☐	6☐
3. 90% or 95% lean ground meat or turkey	1☐	2☐	3☐	4☐	5☐	6☐
4. Turkey burgers or veggie burgers	1☐	2☐	3☐	4☐	5☐	6☐
5. Foods made with oil instead of butter or lard	1☐	2☐	3☐	4☐	5☐	6☐
6. Foods made with a small amount of cheese	1☐	2☐	3☐	4☐	5☐	6☐

Please respond to each item by marking ONE box per row with an X.

B. Assuming you want to, how confident are you that you could do each of the following starting this week and continuing for at least 1 month?

How confident are you that you could…	Not at all confident	Not very confident	Neutral	Very confident	Extremely confident
7. **Choose baked (not regular, fried) plantain or potato chips** instead of regular plantain or potato chips	1☐	2☐	3☐	4☐	5☐
8. **Choose chicken that is roasted, baked, broiled, boiled, or grilled** instead of fried chicken?	1☐	2☐	3☐	4☐	5☐
9. **Choose 90% or 95% lean ground meat or turkey** instead of less lean, 75% to 85% lean, ground meat or turkey?	1☐	2☐	3☐	4☐	5☐
10. **Choose turkey burgers or veggie burgers** instead of hamburgers or cheeseburgers?	1☐	2☐	3☐	4☐	5☐
11. **Choose foods cooked with oil** instead of butter or lard?	1☐	2☐	3☐	4☐	5☐
12. **Choose foods made with a small amount of cheese** instead of foods with a lot of cheese?	1☐	2☐	3☐	4☐	5☐

Section 4. Dietary Sugar Intake (12 questions)

Please respond to each item by marking ONE box per row with an X.

A. Next, how much you like or dislike each type of food or drink listed below?

	Strongly dislike	Dislike	Neutral	Like	Strongly like	Never tried it
1. Hot cereal without any type of added sugar like plain oatmeal or plain cream of wheat	1☐	2☐	3☐	4☐	5☐	6☐
2. Fresh or frozen fruit as a sweet, treat, or dessert	1☐	2☐	3☐	4☐	5☐	6☐
3. Plain yogurt (with or without adding fruit yourself) or "light" yogurt	1☐	2☐	3☐	4☐	5☐	6☐
4. Water, sparkling water, or water mixed with slices of fruits or vegetables	1☐	2☐	3☐	4☐	5☐	6☐
5. Plain, unsweetened, milks (cow, almond, soy, rice)	1☐	2☐	3☐	4☐	5☐	6☐
6. Salad dressings and sauces (such as tomato sauce) with low amounts of added sugar or that you make yourself	1☐	2☐	3☐	4☐	5☐	6☐

Please respond to each item by marking ONE box per row with an X.

B. Assuming you want to, how confident are you that you could do each of the following starting this week and continuing for at least 1 month?

How confident are you that you could…	Not at all confident	Not very confident	Neutral	Very confident	Extremely confident	Does not apply
7. **Choose hot cereal without any type of added sugar like plain oatmeal or plain cream of wheat** instead of hot cereal with added sugar like Maple and Brown Sugar Oatmeal, Cream of Wheat with added sugar?	1☐	2☐	3☐	4☐	5☐	6☐
8. **Choose fresh or frozen fruit as a sweet, treat, or dessert** instead of cookies, candy, cakes, or pastries?	1☐	2☐	3☐	4☐	5☐	6☐
9. **Choose plain yogurt (with or without adding fruit) or "light" yogurt** instead of ice cream, yogurt with more added sugar, milkshakes?	1☐	2☐	3☐	4☐	5☐	6☐
10. **Choose water, sparkling water, or water mixed with slices of fruits or vegetables** instead of drinks with added sugar such as flavored waters, sodas, juices, or energy drinks?	1☐	2☐	3☐	4☐	5☐	6☐
11. **Plain, unsweetened, milks (cow, almond, soy, rice)** instead of drinks with added sugar such as chocolate or vanilla milks, flavored waters, sodas, juices, or energy drinks?	1☐	2☐	3☐	4☐	5☐	6☐
12. **Choose salad dressings and sauces (such as tomato sauce) with low amounts of added sugar or that you make yourself** instead of salad dressings and sauces with higher amounts of added sugar?	1☐	2☐	3☐	4☐	5☐	6☐

Preferences and Self-Efficacy of Diet and Physical Activity Behaviors Questionnaire for Latina Women – Short version 1, 47 questions

Behavior	Questions
Physical Activity	13
Fruit and Vegetables	10
Dietary Fat	12
Dietary Sugar	12
Total	47

Preferences and Self-Efficacy of Diet and Physical Activity Behaviors Questionnaire for Latina Women – Shortened version 2, 41 questions

Section 1. Physical Activity (13 questions)

We will ask you about your physical activity. Please respond to each item by marking ONE box per row with an X.

A. Please indicate which types of physical activity you like to do.

	Strongly dislike	Dislike	Neutral	Like	Strongly like	Never tried it
1. Dancing in a group setting	1☐	2☐	3☐	4☐	5☐	6☐
2. Playing with children with vigorous effort	1☐	2☐	3☐	4☐	5☐	6☐
3. Doing vigorous activities at the gym	1☐	2☐	3☐	4☐	5☐	6☐
4. Walking very quickly and/or uphill for leisure	1☐	2☐	3☐	4☐	5☐	6☐
5. Walking at a brisk pace to get from place to place	1☐	2☐	3☐	4☐	5☐	6☐
6. Bicycling using vigorous effort for leisure	1☐	2☐	3☐	4☐	5☐	6☐
7. Dancing at home	1☐	2☐	3☐	4☐	5☐	6☐
8. Doing housework with moderate effort	1☐	2☐	3☐	4☐	5☐	6☐

Definitions. For the following questions,

- **Moderate to vigorous** physical activities refer to activities that take moderate to vigorous physical effort and make you breathe somewhat harder or much harder than normal. You may have a difficult time having conversations. *Examples include* walking briskly, running, carrying children upstairs or for distances, and doing housework like carrying grocery bags.
- **"Regular"** physical activity means 2 ½ hours (150 minutes) per week. If we think about it on a daily basis, it would be 20 minutes every day or 30 minutes 5 times per week.

Please respond to each item by marking ONE box per row with an X.

B. Please rate your confidence in participating in regular moderate to vigorous physical activity over the next month.

**How confident are you that you could participate in regular moderate to vigorous physical activity over the next month?**	**Not at all confident**	**Not very confident**	**Neutral**	**Very confident**	**Extremely confident**
9. When you can't notice any improvements in your fitness	1☐	2☐	3☐	4☐	5☐
10. When you have many other demands on your time	1☐	2☐	3☐	4☐	5☐
11. When you feel a little stiff or sore	1☐	2☐	3☐	4☐	5☐
12. When the weather is bad	1☐	2☐	3☐	4☐	5☐
13. When you have to get up early to do physical activity, even on weekends	1☐	2☐	3☐	4☐	5☐

Section 2. Fruits and Vegetable Intake (10 questions)

Please respond to each item by marking ONE box per row with an X.

A. For each of the fruits and vegetables listed below, how much you like or dislike each type of fruit or vegetable?

	Strongly dislike	Dislike	Neutral	Like	Strongly like	Never tried it
1. Green cabbage	1☐	2☐	3☐	4☐	5☐	6☐
2. Spaghetti squash	1☐	2☐	3☐	4☐	5☐	6☐
3. Kale	1☐	2☐	3☐	4☐	5☐	6☐
4. Tomatoes, tomato paste, canned tomatoes	1☐	2☐	3☐	4☐	5☐	6☐
5. Butternut squash	1☐	2☐	3☐	4☐	5☐	6☐
6. Chard	1☐	2☐	3☐	4☐	5☐	6☐

Please respond to each item by marking ONE box per row with an X.

B. Assuming that you want to, how confident are you that you could do each of the following starting this week and continuing for at least 1 month?

	Not at all confident	Not very confident	Neutral	Very confident	Extremely confident	Does not apply
7. Eat a healthy snack, like a fruit or a vegetable, when you're really hungry?	1☐	2☐	3☐	4☐	5☐	6☐
8. Eat healthy foods, like fruits or vegetables, when you are tired?	1☐	2☐	3☐	4☐	5☐	6☐
9. Eat fruits and vegetables when your family and friends are eating junk foods like chips, cookies, or candy?	1☐	2☐	3☐	4☐	5☐	6☐
10. Snack on fruits and vegetables rather than on junk foods while watching TV?	1☐	2☐	3☐	4☐	5☐	6☐

Section 3. Dietary Fat Intake (10 questions)

Please respond to each item by marking ONE box per row with an X.

A. Next, how much you like or dislike each type of food or drink listed below?

	Strongly dislike	Dislike	Neutral	Like	Strongly like	Never tried it
1. Baked (not regular, fried) plantain or potato chips	1☐	2☐	3☐	4☐	5☐	6☐
2. Chicken that is roasted, baked, broiled, boiled, or grilled	1☐	2☐	3☐	4☐	5☐	6☐
3. 90% or 95% lean ground meat or turkey	1☐	2☐	3☐	4☐	5☐	6☐
4. Pork and/or beef without visible (white) fat	1☐	2☐	3☐	4☐	5☐	6☐
5. Foods made with oil instead of butter or lard	1☐	2☐	3☐	4☐	5☐	6☐

Please respond to each item by marking ONE box per row with an X.

B. Assuming you want to, how confident are you that you could do each of the following starting this week and continuing for at least 1 month?

**How confident are you that you could…**	**Not at all confident**	**Not very confident**	**Neutral**	**Very confident**	**Extremely confident**
6. **Choose baked (not regular, fried) plantain or potato chips** instead of regular plantain or potato chips	1☐	2☐	3☐	4☐	5☐
7. **Choose chicken that is roasted, baked, broiled, boiled, or grilled** instead of fried chicken?	1☐	2☐	3☐	4☐	5☐
8. **Choose 90% or 95% lean ground meat or turkey** instead of less lean, 75% to 85% lean, ground meat or turkey?	1☐	2☐	3☐	4☐	5☐
9. **Choose pork and/or beef without visible (white) fat** instead of pork and/or beef with visible fat?	1☐	2☐	3☐	4☐	5☐
10. **Choose foods cooked with oil** instead of butter or lard?	1☐	2☐	3☐	4☐	5☐

Section 4. Dietary Sugar Intake (8 questions)

Please respond to each item by marking ONE box per row with an X.

A. Next, how much you like or dislike each type of food or drink listed below?

	Strongly dislike	Dislike	Neutral	Like	Strongly like	Never tried it
1. Fresh or frozen fruit as a sweet, treat, or dessert	1☐	2☐	3☐	4☐	5☐	6☐
2. Water, sparkling water, or water mixed with slices of fruits or vegetables	1☐	2☐	3☐	4☐	5☐	6☐
3. Plain, unsweetened, milks (cow, almond, soy, rice)	1☐	2☐	3☐	4☐	5☐	6☐
4. Salad dressings and sauces (such as tomato sauce) with low amounts of added sugar or that you make yourself	1☐	2☐	3☐	4☐	5☐	6☐

Please respond to each item by marking ONE box per row with an X.

B. Assuming you want to, how confident are you that you could do each of the following starting this week and continuing for at least 1 month?

How confident are you that you could…	Not at all confident	Not very confident	Neutral	Very confident	Extremely confident	Does not apply
5. **Choose fresh or frozen fruit as a sweet, treat, or dessert** instead of cookies, candy, cakes, or pastries?	1☐	2☐	3☐	4☐	5☐	6☐
6. **Choose water, sparkling water, or water mixed with slices of fruits or vegetables** instead of drinks with added sugar such as flavored waters, sodas, juices, or energy drinks?	1☐	2☐	3☐	4☐	5☐	6☐
7. **Plain, unsweetened, milks (cow, almond, soy, rice)** instead of drinks with added sugar such as chocolate or vanilla milks, flavored waters, sodas, juices, or energy drinks?	1☐	2☐	3☐	4☐	5☐	6☐
8. **Choose salad dressings and sauces (such as tomato sauce) with low amounts of added sugar or that you make yourself** instead of salad dressings and sauces with higher amounts of added sugar?	1☐	2☐	3☐	4☐	5☐	6☐

Preferences and Self-Efficacy of Diet and Physical Activity Behaviors Questionnaire for Latina Women – Short version 2, 41 questions

Behavior	Questions
Physical Activity	13
Fruit and Vegetables	10
Dietary Fat	10
Dietary Sugar	8
Total	41
